# Rehabilitation of lexical and semantic communicative impairments: An overview of available approaches

**DOI:** 10.1590/S1980-57642014DN83000011

**Published:** 2014

**Authors:** Fabíola Schwengber Casarin, Laura Branco, Natalie Pereira, Renata Kochhann, Gigiane Gindri, Rochele Paz Fonseca

**Affiliations:** 1 Graduate Psychology Program, Department of Psychology, Pontifical Catholic University of Rio Grande do Sul - PUCRS, Porto Alegre, RS, Brazil.; 2 CAPES PhD Scholarship. Department of Psychology, PUCRS.; 3 BPA/PUCRS Undergraduate Scholarship, Department of Psychology - PUCRS.; 4 CAPES Master’s Scholarship, Department of Psychology- PUCRS.; 5 PhD. CAPES Postdoctoral Scholarship (DOCFIX), Department of Psychology - PUCRS.; 6 PhD. Speech Therapist from Hospital Nossa Senhora da Conceição, Porto Alegre, RS, Brazil.; 7 PhD. Adjunct Professor, Department of Psychology - PUCRS.

**Keywords:** rehabilitation of speech and language disorders, language therapy anomia, communication

## Abstract

Lexical-semantic impairments are common consequences of acquired neurological damage. However, little is known about the benefits of existing treatment methods for this type of language impairment.

**Objective:**

To evaluate current research into lexical-semantic interventions for adults with dementia, TBI or stroke.

**Methods:**

The PubMed, PsycInfo and SCOPUS databases were searched for studies related to rehabilitation, neurological conditions, communicative and lexical-semantic skills published between 2004 and 2014.

**Results:**

Twenty-eight of the 453 abstracts found were selected for the review based on the PRISMA method. Most of the studies described treatments for anomia. Semantic tasks were the most commonly used, followed by phonological and gestural strategies. Interventions were individual and involved formal tasks, although the number, frequency and duration of sessions varied between studies.

**Conclusion:**

Although lexical-semantic interventions lead to improvements in language abilities, they are still poorly described in the literature, and must be further investigated in terms of their efficacy, effectiveness and long-term effects.

## Abstract (pt)

Déficits em processamento comunicativo léxico-semântico podem acompanhar quadros neurológicos. Mas, pouco se sabe do efeito terapêutico de intervenções léxico-semânticas.

**Objetivo:**

Esta revisão sistemática caracterizou estudos com este tipo de intervenção, em pacientes adultos com demência, pós TCE e pós AVC.

**Métodos:**

Analisaram-se estudos publicados entre 2004 e 2014 nas bases de dados PubMed, PsycInfo e SCOPUS, com descritores dos construtos reabilitação, quadro neurológico adquirido, comunicação e componente léxico-semântico.

**Resultados:**

Dos 453 estudos encontrados, 28 foram selecionados, seguindo método prisma. Destes, a maioria apresentava terapia para anomia. As técnicas mais empregadas foram de processamento léxico-semântico, seguida pelo fonológico e gestual. Em sua maioria, a intervenção ocorreu na modalidade individual, com uso de tarefas formais, apesar da variabilidade do número, frequência e duração das sessões.

**Conclusão:**

Os tratamentos propiciaram melhora no desempenho na avaliação pós-intervenção. No entanto, há limitações da intervenção léxico-semântica, especialmente quanto à descrição teórica das técnicas e faltam estudos de eficácia, de efetividade, e de acompanhamento.

## INTRODUCTION

Lexical-semantic processing refers to language comprehension and expression at the word level.^1,2^ Much of the knowledge regarding these processes has been obtained through the study of lexical-semantic impairments in populations with acquired neurological damage, especially left hemisphere strokes^3^ and traumatic brain injury (TBI).^4^ Significant research has also been conducted into the lexical-semantic abilities of individuals with neurodegenerative conditions such as dementia.^5^ These studies have been particularly relevant for primary progressive aphasia (PPA),^6^ whose subtypes are classified based on the nature of the language impairments present.^7^

Lexical and semantic impairments may cause a variety of linguistic and cognitive alterations, the most common of which is anomia, consisting of difficulties in naming or specific word recall.^8,9^ Although the left hemisphere (LH) is traditionally considered dominant for language processing, lexical and semantic deficits have also been reported in patients with right hemisphere damage (RHD). Such impairments are especially evident in semantic judgment and association tasks,^10^ in the metaphorical understanding of ambiguous language, as well as in verbal fluency tasks, in which individuals with RHD tend to evoke more abstract, less prototypical and more uncommon words than control subjects.^11,12^

Language is an especially important domain in neuropsychological assessment, not least due to its bidirectional relationship with other cognitive functions such as the different types of attention and memory, and executive functions. Additionally, language is also the means by which most neurocognitive instruments evaluate their target constructs. As such, language development has been the focus of several neuropsychological studies, which have found, for instance, that it may be influenced by factors such as age, education and gender even in healthy individuals.^13,14^ Associations among cognitive skills, language development, cognitive stimulation and lifestyle factors must always be considered during neuropsychological evaluation and rehabilitation. Therefore, in an attempt to ensure the comprehensive assessment of language skills, several instruments have been made available for the evaluation of phonetic-phonological, syntactic-semantic and pragmatic-discursive features of spoken language, especially in patients with acquired brain injury.^15^

Given the high prevalence of anomia as a symptom of neurological disorders, several interventions have also been developed to assist with the compensation or attenuation of naming difficulties based on different theoretical models.^16^ Common examples of such interventions include semantic feature analysis (SFA);^17-19^ and semantic treatments,^20,21^ although interventions have also been developed based on phonological^22^ and gestural approaches,^23^ or semantic priming.^9^ More recently, strategies involving cognitive-linguistic and communicative therapy approaches^24^ and sentence generation^25^ have also been described. Interestingly, although several techniques have been developed for the rehabilitation of patients with classic aphasia following LHD,^19^ few empirical studies, reviews or meta-analyses have evaluated interventions for communicative impairments, and only general guidelines are available on the topic.^26^

Literature reviews have proved to be a useful tool for the evaluation of the effects of different interventions on lexical-semantic processing in conditions such as anomic aphasia^16^ or semantic variant PPA.^21^ Prosodic, discursive and pragmatic impairments, as well as the treatment needs and intervention guidelines for adults with RH lesions have also been reviewed by Ferré et al.^10^ Therefore, in light of the important contributions made to the literature by other reviews in the past, the aim of the present study was to use this method to describe and evaluate current research on lexical-semantic interventions, focusing on the objectives, methods and results of the studies performed on the topic. This study also entailed a careful and detailed analysis of the methods used by different lexical-semantic interventions, such as the number, frequency and duration of rehabilitation sessions, the number of therapists involved, the use of individual *versus* group interventions and formal or ecological tasks, as well as the minimal performance criteria applied. This information can help guide future practice and research into lexical-semantic rehabilitation. Our aim can be translated into the following research question: What methodological variables (sample, assessments and interventions), linguistic features and outcomes are evaluated in existing lexical-semantic rehabilitation programs for patients with dementia, TBI or stroke?

**Literature review.** The present review was based on the PRISMA guidelines,^27^ and performed in May 2014. The PubMed, Psycinfo and Scopus databases were searched for articles regarding lexical and semantic rehabilitation published in the past 10 years. Since interventions for patients with anomia have a long history and are among the most frequently discussed in the literature, we decided to focus on more recent studies in the area. Since most of the rehabilitation programs developed over the past ten years have been based on theoretical models and on assessment methods established in the 1990s and 2000s, we focused on studies published within this time period.^28^ Different sets of keywords were used to retrieve articles related to each of the four main constructs evaluated in the present review (rehabilitation, acquired neurological damage, communicative skills and lexical-semantic abilities). The following keywords were used to retrieve articles regarding rehabilitation interventions: "*rehabilitation", "readaptation", "reeducation", "training", "intervention", "treatment", "therapy", "functional recovery" and "remediation".* Articles relating to neurological conditions were identified using the following terms: "*stroke", "cerebrovascular disease", "cerebrovascular accident", "right hemisphere damage", "left hemisphere damage", "lesion studies", "brain injury", "brain damage", "traumatic brain injury", "closed head injury"* and *"dementia"*. Investigations into communicative skills were retrieved using the keywords "*communication", "linguistic", "language", "communication", "communicative"* and* "aphasia"*. Lastly, studies of lexical-semantic processing were retrieved using the terms "*lexical", "lexicon", "semantics", "verbal fluency", "lexical-semantic", "word level", "category fluency", "categorical fluency", "letter fluency", "phonemic fluency"* and *"semantic fluency".*

The abstracts retrieved were screened based on the following inclusion criteria:^1^ empirical study,^2^ involvement of at least one adult with an acquired neurological condition,^3^ description of lexical-semantic rehabilitation procedures,^4^ presence of pre- and post-intervention assessments, and^5^ publication in English, French, Spanish or Portuguese. All abstracts were examined by two independent judges, and discrepancies were settled by a third reviewer. This process resulted in the exclusion of 419 abstracts (n=334 focused on language assessment only and n=30 on general cognitive rehabilitation, n=23 were literature reviews, n=10 were duplicates, n=10 focused on medication effects, n=5 described attention and memory rehabilitation strategies, n=3 dealt with motor interventions only, n=2 evaluated the effects of music therapy, n=1 assessed the effects of psychotherapy, and =1 described occupational therapy interventions).

**Figure 1:**
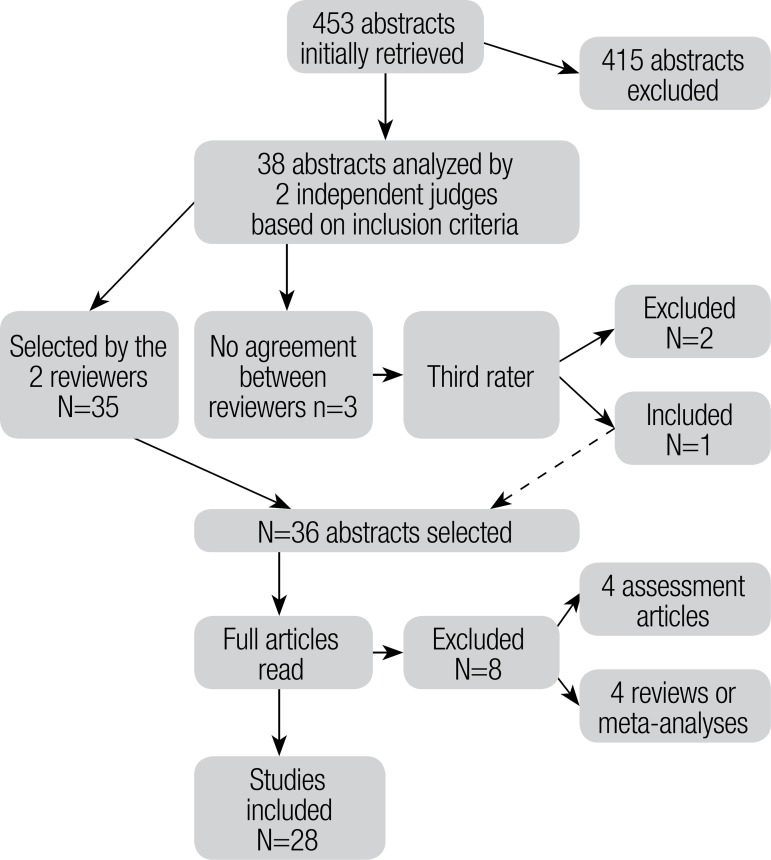
Flow diagram of the article selection process.

As can be seen in Figure 1, after exclusion criteria were applied, only 38 of the initially retrieved abstracts remained. These abstracts were reevaluated by the two judges, who disagreed on the inclusion of three articles. The third reviewer decided on the inclusion of one article, while the other two were excluded. The remaining 36 articles were read in full, resulting in the further exclusion of four papers which consisted of meta-analyses and literature reviews, as well as another four which dealt exclusively with language assessment. Consequently, a total of 28 articles were included the present review.

The objectives, sample, theoretical basis of the intervention, language assessment instruments and results described in each of the studies included are described in Table 1. Further details regarding the interventions themselves are shown in Table 2, which describes the number, frequency and duration of the rehabilitation sessions conducted, as well as the number of therapists involved in the treatment, the use of group *versus* individual interventions and formal *versus* ecological tasks, as well as the establishment of minimal performance criteria.

**Table 1:** Characterization of the objectives, sample, type of intervention, assessment procedures and results of studies involving lexical-semantic rehabilitation.

Reference	Objective	Sample	Intervention Method	Assessment		Results
				Timepoints	Constructs assessed	
(^29^) Henry, Rising, DeMarco, Miller, Gorno-Tempini & Beeson (2013)	To describe the implementation and outcomes of language treatment based on the recruitment of spared cognitive and neural systems in patients with PPA.	2 patients (n=1 with semantic variant PPA and n=1 with logopenic PPA).	Lexical Retrieval Cascade Treatment: training of lexical retrieval strategies to engage and strengthen residual semantic, orthographic and semantic knowledge.	1) Baseline 2) Maintenance 3) 1-month follow-up 4) 3-month follow-up	Speech, language, cognition.	Both participants improved their ability to name target nouns. Gains were maintained at follow-up, and retrieval strategy was generalized to untrained items. Functional benefits were reported.
(^30^) Van Hees, McMahon, Angwin, Zubicaray, Read & Copland (2014)	To examine the neural mechanisms responsible for treatment-induced improvement in anomia.	8 patients with aphasia.	SFA and PCA.	1) Baseline 2) Post-treatment	Language.	Pre-treatment activity in the left caudate was associated with the success of SFA treatment. Success of PCA was correlated with post-treatment activation in the left supramarginal gyrus and right precuneus.
(^31^) Savage, Ballard, Piguet, & Hodges (2013)	To evaluate the use of a home-based word-learning method for the treatment of naming impairments in semantic variant PPA.	4 patients with semantic variant PPA.	Picture-word pairing.	1) Baseline 2) Weekly assessments during treatment 3) Follow-up 1 4) Follow-up 2	Visuospatial ability, attention, executive functions, language.	All patients displayed significant improvements in the ability to name target items. Gains were maintained at follow-up.
(^32^) Agostini, Garzon, Benavides-Varela, De Pellegrin, Bencini, Rossi, Mancuso, Turolla, Meneghello & Tonin (2014)	To explore the feasibility of telerehabilitation as compared to conventional face-to-face interventions for naming impairments.	5 patients with aphasia.	Telerehabilitation and conventional face-to-face therapy.	1) Baseline 1 2) Baseline 2 3) Post-treatment 1 4) Post-treatment 2 5) Follow-up	Language, attention, intelligence.	Telerehabilitation and conventional treatment produced similar results for patients with aphasia.
(^33^) van Hees, Angwin, McMahon & Copland (2013)	To investigate the relative effects of SFA and PCA strategies in patients with aphasia.	8 patients with aphasia.	SFA and PCA.	1) Baseline 2) Probe 3) Follow-up	Language.	7 participants improved following PCA (6 maintained improvement at follow-up) and 4 improved following SFA (3 maintained improvement at follow-up).
(^34^) Arroyo-Anlló, Ingrand & Gil (2012)	To determine whether patients with Alzheimer’s Disease could develop semantic skills using a cognitive procedural task.	20 patients with mild Alzheimer’s Disease.	Computerized procedural semantic categorization task.	1) Baseline 2) Post-treatment	Language, general cognitive abilities.	Patients with Alzheimer’s disease were able to acquire semantic skills by repeated exposure to the categorization task.
(^35^) Ferguson, Evans & Raymer (2012)	To compare the effectiveness of intention and pantomime gesture treatment for patients with chronic aphasia.	4 patients with chronic aphasia.	Intention and gestural training.	1) Baseline 2) Post-treatment	Language.	Intention gesture training resulted in improved naming of target nouns, while pantomime gesture training led to improved non-verbal communication skills across trained and untrained nouns.
(^36^) Jelcic, Cagnin, Meneghello, Turolla, Ermani & Dam (2012)	To investigate the effect of lexical-semantic stimulation on verbal communication and episodic memory in early Alzheimer’s Disease.	40 patients with early Alzheimer’s Disease.	Lexical semantic stimulation (treatment) or unstructured cognitive stimulation (control).	1) Baseline 2) Post-treatment 3) 3-month follow-up 4) 6-month follow-up (experimental group only)	Language, episodic memory, working memory, attention, executive functions.	Lexical-semantic stimulation led to improvements in cognition and memory, which remained during follow-up.
(^3^) Raymer, Mc Hose, Smith, ImanAmbrose & Casselton (2012)	To compare the effects of errorless naming treatment and gestural facilitation for the treatment of naming impairments in patients with aphasia.	8 patients with aphasia after left-hemisphere stroke.	Errorless naming treatment and Gestural Facilitation Training.	1) Baseline 2) Post-intervention 3) 1-month follow-up	Language.	Both methods led to similar improvements in naming ability.
(^5^) Noonan, Pryer, Jones, Burns & Lambon Ralph (2012)	To contrast the effects of Errorless and Errorful Learning treatment of naming impairments in patients with Alzheimer’s Disease.	8 adults with mild to moderate Alzheimer’s Disease and severe anomia.	Errorless Learning or Errorful Learning.	1) Baseline 2) 1-week follow-up 3) 5-week follow-up	Language, episodic memory, semantic memory, attention, executive functions.	Both techniques led to improvements in the naming of trained and untrained items. Benefits remained during follow-up.
(^37^) Jokel & Anderson (2012)	To evaluate the effects of Errorless Learning versus Errorful Learning and passive versus active learning in semantic variant PPA.	7 patients with mild to moderate semantic variant PPA.	1) Errorless learning – active 2) Errorless learning- passive 3) Errorful learning – active 4) Errorful learning - passive	1) Baseline 2) Post-intervention 3) 1-month follow-up 4) 3-month follow-up	Language, episodic memory, working memory, executive functions, visuospatial function.	Errorless Learning was more effective than Errorful Learning for the treatment of naming impairment. No differences were observed between active and passive learning.
(38) Beeson, King, Bonakdarpour, Henry, Cho & Rapcsak (2011)	To evaluate the effects of intensive naming treatment in a patient with early logopenic PPA.	1 patient with logopenic PPA.	Item-naming for six semantic categories.	1) Baseline 2) During training 3) Post-intervention 4) 3-week follow-up 5) 4-month follow-up 6) 6-month follow-up	Speech, language comprehension and expression, non-verbal cognitive skills, behavioral competencies.	Treatment led to improved lexical retrieval for trained and untrained items.
(^39^) Kurland & Falcon (2011)	To evaluate the effects of bilingual semantic naming treatment and the effects of cognates on cross-linguistic generalization.	1 bilingual patient with severe aphasia following hemorrhagic left-hemisphere stroke.	Constraint-induced Language Therapy (CILT).	1) Baseline 2) Post-treatment 1 (Spanish) 3) Post-treatment 2 (English) 4) Post-treatment 3 (Bilingual therapy)	Language.	Improved naming and auditory comprehension were observed following treatment. Some cross-linguistic effects were observed, although cognates interfered with target word selection.
(^40^) Kieran, & Iakupova (2011)	To evaluate aphasia in bilingual patients, and examine how these individuals re-acquire lexical-semantic proficiency as a result of therapy.	2 bilingual patients with aphasia following stroke.	SFA	1) Baseline 2) Post-treatment	Language.	Patient showed significant improvements in both the trained and untrained languages.
(^41^) Edmonds & Babb (2011)	To evaluate the effects of Verb Network Strengthening Treatment in patients with moderate to severe aphasia.	2 patients with aphasia.	Verb Network Strengthening Treatment (VNeST).	1) Baseline 2) 1-month follow-up 3) 5-month follow-up	Language, speech apraxia, attention, executive functions, visuospatial function, memory.	One patient exhibited improvement on all generalization measures, while the other participant exhibited more limited generalization.
(^6^) Croft, Marshall, Pring & Hardwick (2011)	To investigate whether bilingual patients with aphasia respond to monolingual treatment strategies, and whether languages respond differently to therapy and to assess cross-linguistic generalization.	5 bilingual patients with aphasia.	Treatment in English and Bengali, involving semantic and phonological tasks.	1) Baseline 2) Post-treatment 3) Follow-up	Language.	Monolingual treatments may benefit bilingual patients with aphasia, and improve performance in both L1 and L2.
(^42^) Yeung, Law & Yau (2009)	To examine associations between treatment generalization and executive control.	5 patients with anomia following a brain lesion.	Naming treatment with phonological cues.	1) Baseline 2) Post-treatment	Auditory discrimination, repetition, naming, visuospatial processing, reading, verbal and non-verbal semantic processing, attention, executive functions.	All participants demonstrated improvement in naming treated items. Inhibitory control was positively associated with treatment generalization.
(^24^) Jong-Hagelstein et al. (2010)	To evaluate the efficacy of cognitive-linguistic and communicative treatment in aphasia after stroke.	75 patients with aphasia following left-hemisphere strokes.	Cognitive-linguistic and communicative treatment.	1) Baseline 2) Post-treatment	Language.	Both treatments led to significant improvements in communicative skills.
(^22^) Kendall, Rosenbek, Heilman, Conway, Klenberg, Rothi & Nadeau (2008)	To investigate the effects of phonological treatment for anomia.	10 patients with aphasia following left-hemisphere strokes.	Phoneme-based treatment.	1) Baseline 2) Post-treatment	Language.	Positive treatment effects were observed in confrontation naming, phonologic production and nonword repetition. Generalization to discourse production was observed. Effects remained during follow-up.
(^21^) Henry, Beeson & Rapcsak, (2008)	To assess the effects of semantic treatment in progressive and stroke-induced aphasia.	2 patients with progressive aphasia and 1 patient with stroke-induced aphasia.	Generative naming for selected semantic categories.	1) Baseline 2) 3-week follow-up 3) 4-month follow-up	Language, memory.	Treatment resulted in both immediate and long-term benefits for all patients in the sample.
(^23^) Raymer, Singletary, Rodriguez, Ciampitti, Heilman & Rothi (2006)	To evaluate the effects of gestural verb training in patients with aphasia.	9 patients with aphasia following left-hemisphere strokes.	Gestural verb training.	1) Baseline 2) Post-treatment	Naming, speech.	Treatment led to similar improvements in noun and verb naming.
(^25^) Raymer & Cohen (2006)	To explore the effects of word-retrieval training in a sentence context for both nouns and verbs.	1 patient with stroke-induced aphasia and 1 patient with nonfluent transcortical motor aphasia and moderate apraxia of speech.	Sentence-embedded word retrieval training protocol using action pictures paired with oral reading of corresponding sentences.	1) Baseline 2) Post-treatment	Language comprehension and expression at the word and sentence level.	Both participants demonstrated positive effects of treatment. Participant 2 responded better to the sentence-level training than participant 1.
(^9^) Law, Wong, Sung & Hon (2006)	To assess the efficacy of a treatment combining semantic feature analysis and semantic priming.	3 patients with anomia following left-hemisphere strokes.	SFA and semantic priming.	1) Baseline 2) During treatment 3) Post-treatment	Naming.	Treatment gains and generalization were observed in two participants. The patient with severe semantic impairment did not benefit from treatment.
(^43^) Edmonds & Kiran (2006)	To assess the cross-linguistic generalization of naming treatment in bilingual patients with aphasia.	3 bilingual patients with aphasia.	SFA	1) Baseline 2) 1-month follow-up 3) 4-month follow-up	Language.	Treatment in the non-dominant language may facilitate cross-linguistic generalization of treatment gains.
(^44^) Jokel , Rochon & Leonard (2006)	To present the case study of a patient with semantic variant PPA who received home-based rehabilitation.	1 patient with semantic variant PPA and anomia.	Naming treatment with phonological and semantic cues.	1) Baseline 2) Post-treatment 3) 1-month follow-up 4) 6-month follow-up	Language.	Treatment delayed the progression of language impairments.
(^45^)Beeson & Egnor (2005)	To assess the effects of a treatment involving both spoken and written naming in patients with lexical-semantic impairments.	2 patients with stroke-induced aphasia.	Copy and Recall Treatment (CART) and spoken repetition of selected stimuli.	1) Baseline 2) Post-treatment	Language.	Treatments involving combined written and spoken naming led to significant improvements in patients with residual phonological abilities.
(^46^) Bretenstein, Kamping, Jansen, Schomacher & Knecht (2004)	To assess whether patients with aphasia can learn new vocabulary by intense frequency of exposure alone.	1 patient with fluent aphasia and 1 patient with non-fluent aphasia.	Associative Learning Task.	1) Baseline 2) After each session	Language, naming, verbal memory, visuospatial memory, semantic verbal fluency.	Word re-learning in aphasia benefitted from maximizing the frequency of exposure and the use of massed practice.
(^20^) Doesborgh Sandt-Koenderman, Dippel, van Harskamp, Koudstaal & Visch-Brink, (2003)	To investigate the effects of semantic treatment in a randomized controlled trial.	55 adults with lexical and semantic impairment following left-hemisphere strokes.	Semantic and phonological treatment.	1) Baseline 2) Post-treatment	Language.	Semantic and phonological treatments led to similar improvements in communicative skills.

PPA: primary progressive aphasia; SFA: semantic feature analysis; PCA: phonological components analysis.

**Table 2:** Intervention methods.

Reference	Number of sessions	Weekly frequency	Session duration	Number of therapists	Mode of intervention (group or individual)	Type of task used (formal or ecological)	Minimum performance requirements
(^29^) Henry, Rising, DeMarco, Miller, Gorno-Tempini & Beeson (2013).	P1: 8 sessions - 4 weeks of treatment (8 hours) and 20 hours of homework. P2: 6 sessions - 8 weeks (6 hours) and 18 hours of homework.	2 a week	1 h	Not specified	Individual	Formal: Semantic self-cue, Orthographic self-cue, Phonemic self-cue, Oral reading, Repetition, Semantic plausibility, Judgments and Recall.	80% or greater accuracy on a single set in a single session or in two consecutive sessions.
(^30^) Van Hees, McMahon, Angwin, Zubicaray, Read & Copland (2014)	12	Not specified	Not specified	Not specified	Individual	Formal: Figure naming	Not specified
(^31^) Savage, Ballard, Piguet, & Hodges (2013)	1 assessment session a week + daily practice at home	Daily	Not specified	Not specified	Individual	Ecological: Word-picture pairing, sentence generation.	Not specified
(^32^) Agostini, Garzon, Benavides-Varela, De Pellegrin, Bencini, Rossi, Mancuso, Turolla, Meneghello & Tonin (2014)	16	Not specified	Not specified	Not specified	Individual	Formal: Confrontation-naming with progressive phonemic cues if no response given or response incorrect	Not specified
(^33^) van Hees, Angwin, McMahon & Copland (2013)	12	3 a week	45-60 min (patients with mild to moderate aphasia) 60-90 min (patients with severe aphasia)	Not specified	Individual	Formal: Naming and description of semantic and phonological features	100% naming accuracy on treatment set
(^34^) Arroyo-Anlló, Ingrand & Gil (2012)	Not specified	Not specified	Not specified	Not specified	Individual	Formal: Procedural semantic categorization task	Not specified
(^35^) Ferguson, Evans & Raymer (2012)	Not specified	2 to 3 a week	Not specified	Not specified	Individual	Formal: Paired verbal production of target nouns paired with intention or pantomime gestures.	90% accuracy across 3 sessions, or 10 treatment sessions completed
(^36^) Jelcic, Cagnin, Meneghello, Turolla, Ermani & Dam (2012)	24	2 a week	60 min	1	Groups of 4 patients	Formal: Lexical semantic stimulation, or unstructured cognitive stimulation.	Not specified
(^3^) Raymer, Mc Hose, Smith, ImanAmbrose & Casselton (2012)	40, divided into 2 phases	2 to 3 a week	60 min	Not specified	Individual		90% accuracy on daily probes, or 20 sessions completed
(^5^) Noonan, Pryer, Jones, Burns & Lambon Ralph (2012)	10	2 a week	40-60 min	Not specified	Not specified	Formal tasks.	Not specified
(^37^) Jokel & Anderson (2012)	96	2 sessions a day, 2 to 3 days a week	30 min	Not specified	Individual	Formal: picture-naming with semantic and phonemic descriptors, question-and-answer exchanges between patient and therapist.	Not specified
(^38^) Beeson, King, Bonakdarpour, Henry, Cho & Rapcsak (2011)	12	6 a week	120 min + 1 h daily homework	Not specified	Individual	Formal. Semantic category naming task.	Not specified
(^39^) Kurland & Falcon (2011)	20	5 a week	150 min	Not specified	Individual	Formal: Intensive naming treatment.	Not specified
(^40^) Kieran, & Iakupova, (2011)	8	4 a week	90 min	1	Individual	Formal: Semantic feature-based treatment.	Not specified
(^41^) Edmonds & Babb (2011)	P1= 45 h P2= 37.5 h	2 a week	120 min	Not specified	Individual	Formal: Verb network strengthening treatment.	80% accuracy
(^6^) Croft, Marshall, Pring & Hardwick (2011)	10	2 a week	60 min	1 English-speaking therapist, 3 bilingual English-Bengali therapists	Individual	Formal: Semantic associate matching, functional questions, naming to definition, semantic feature analysis, repetition, phonological cueing, rhyme judgment, syllable counting, initial phoneme judgment.	Not specified
(^42^) Yeung, Law & Yau (2009)	Not specified	Not specified	Not specified	1	Individual	Formal. Naming treatment with phonemic cues.	80% accuracy in 3 to 4 consecutive sessions
(^24^) Jong-Hagelstein et al. (2010)	45.4 h (33.8h with therapist + 11.6h homework)	2 to 5 h a week	Not specified	Not specified	Not specified	Formal: BOX semantic treatment program or FIKS phonological treatment	Not specified
(^22^) Kendall, Rosenbek, Heilman, Conway, Klenberg, Rothi & Nadeau (2008)	48	4 a week	120 min	2 speech therapists and 1 undergraduate speech therapy student	Individual	Formal: Lindamood Phoneme Sequencing Program	Not specified
(^21^) Henry, Beeson & Rapcsak, (2008)	12	5 a week	90 min	Not specified	Individual	Formal: Semantic tasks (guided retrieval, elaboration within subcategories, production of category exemplars).	Not specified
(^23^) Raymer, Singletary, Rodriguez, Ciampitti, Heilman & Rothi (2006)	20	3 to 4 a week	60 min	Not specified	Individual	Formal: Noun and verb training.	Not specified
(^25^) Raymer & Cohen (2006)	Maximum 10	2 a week	60 min + homework	Not specified	Individual	Formal: Generative production of sentences incorporating target nouns and verbs.	90% accuracy in at least 3 sessions, or 10 sessions completed
(^9^) Law, Wong, Sung & Hon (2006)	20	2 a week	90 a 180 min	1	Individual	Formal: Semantic Feature Analysis and semantic priming.	13 out of 15 items correctly named in 3 consecutive sessions
(^43^) Edmonds & Kiran (2006)	12 to 66	2 a week	120 min	1	Individual	Formal: Semantic tasks	Not specified
(^44^) Jokel , Rochon & Leonard (2006)	21	Daily	30-60 min daily	No therapist	Individual	Formal: Figure naming with semantic and phonological cues.	Not specified
(^45^)Beeson & Egnor (2005)	20	2 a week		1	Individual	Formal: Spoken and written naming, repetition and written copying.	80% accuracy in 2 consecutive sessions
(^46^) Bretenstein, Kamping, Jansen, Schomacher & Knecht (2004)	5	1 to 5 a week	Not specified	No therapist	Individual	Formal: Associative learning task.	Not specified
(^20^) Doesborgh Sandt-Koenderman, Dippel, van Harskamp, Koudstaal & Visch-Brink, (2003)	40 to 60 h	2 to 3 a week	90-180 min	Not specified	Individual	Formal: BOX semantic treatment program or FIKS phonological treatment	Not specified

## RESULTS

Most of the articles retrieved (64.28%) described interventions for naming impairments in patients with aphasia. As can be seen in Table 1, 18 of the 28 studies reviewed involved post-stroke patients with anomia. In four of these studies, the sample was bilingual,^6,39,40,43^ suggesting a growing research interest in the study and rehabilitation of bilingual patients with aphasia. Four additional studies involved patients with semantic variant PPA,^29,31,37,43^ while three reported on individuals with logopenic PPA^21,29,38^ and three involved patients with Alzheimer's disease.^5,34,36^

Most of the studies retrieved (60.71%) described rehabilitation programs involving semantic and phonological interventions.^6,9,20,21,24,29-33,36,38,40,41,43,44,46^ Picture naming with semantic cues was used as a rehabilitation strategy by 39.28% of the studies retrieved, ^6,20,21,24,29-32,38,40,44^ while 17.86% of the articles made use of *Semantic Feature Analysis.*^9,30,33,40,43^ *Lexical Semantic Stimulation (LSS)*,^36^ *Constraint-induced Language Therapy (CILT),*^39^ *Verb Network Strengthening Treatment (VNeST)* (41), while *Semantic Priming*^9^ and *Associative Learning*^46^ were used in one study each. Phonological interventions were described in 17.85% of the studies analyzed,^6,20,33,39,42^ while the remaining 18.51% of the studies used strategies such as *Errorless*^3,5^ and *Errorful Naming Treatments,*^5^ procedural semantic categorization tests,^34^ intention and pantomime gestures^35^ and *Copy and Recall Treatment,* which combines both spoken and written naming.^44^ Only one study involved the use of communicative interventions, which aim to optimize linguistic exchanges using compensatory strategies and residual linguistic skills.^24^ Some studies also performed comparisons between two or more rehabilitation strategies, in an attempt to identify which would be most suitable for the population investigated. Some of the comparisons made by the studies reviewed included face-to-face *versus* telerehabilitation naming treatment,^32^ lexical-semantic stimulation *versus* unstructured cognitive stimulation,^36^ and *errorless naming treatment versus gestural facilitation training.*^3^ In addition to these studies, a further four investigations involved comparisons between multiple rehabilitation approaches.^5,20,24,37^

In all of the studies reviewed, treatment stimuli were selected based on a baseline assessment. Additionally, 21.42% of studies^9,29,33,38,46^ evaluated participant performance during the therapeutic process, 81.48%^3,6,9,22-24,30-34,36-40,42,44-47^ reassessed patients immediately after the training program was completed and 50%^3,5,6,21,31-33,36-39,41,43,44,47^ performed follow-up assessments. Follow-up periods ranged from five days to six months after the end of the intervention. Linguistic competencies were assessed using formal tasks in all of the studies reviewed. Only 25% of the studies evaluated other cognitive components in addition to language, such as attention, memory and executive functioning.

Overall, post-treatment assessments indicated that the interventions led to significant improvements in linguistic performance. As can be seen in Table 2, the interventions involved between five and 96 sessions, performed one to seven days a week. A total of 42.85% of studies involved two weekly sessions.^3,5,6,9,20,23,36,37,41,43,45,46^ The duration of treatment sessions ranged from 30 to 180 minutes, and in 25% of studies, treatment was performed by a single therapist, while 8% of treatments involved more than one speech therapist.^6,22^ The remaining studies did not specify the number of therapists involved in the interventions described. Only one study involved group therapy,^3^ with all remaining studies involving individual interventions and formal rehabilitation methods. Nine of the studies evaluated set mastery criteria for patient accuracy.^3,9,23,24,29,33,35,41,45^.

## DISCUSSION

The aim of the present paper was to review the existing empirical research into lexical-semantic interventions for adult patients with dementia, TBI and stroke. More specifically, this review aimed to answer the following question: What methodological variables (sample, assessments and interventions), linguistic features and outcomes are evaluated in existing lexical-semantic rehabilitation programs in dementia, TBI and stroke?

Most of the studies retrieved focused on the rehabilitation of anomia caused by strokes or neurodegenerative diseases, and involved attempts to search for the most adequate therapeutic interventions for this type of impairment. Given the wide variability in the language impairments observed across neurological conditions, most language rehabilitation research is presented in the form of case studies. Such designs can provide important data as to the effectiveness of different rehabilitation interventions, and allow for the description of interventions which are specifically tailored to the profiles of the patients evaluated.^48^

Although semantic and lexical approaches are still the most commonly used for the treatment of anomia, a growing number of studies have been concerned with comparing the effectiveness of such strategies with that of other techniques, such as phonological interventions.^33^ However, these comparative studies have not yet reached a consensus as to which intervention might be most appropriate for the treatment of anomia.

Other therapeutic approaches, such as the gestural facilitation of naming, were also investigated in the studies reviewed. Raymer et al.,^23^ for instance, found that *gestural facilitation training* was able to help participants recall nouns and verbs, producing beneficial results when combined with *errorless naming treatment.*^35^. In addition to helping with word recall, the use of gestures in a therapeutic setting may encourage patients to make greater use of non-verbal strategies in daily communication, increasing their communicative competence and providing an alternative means of expression for use when word recall is impaired.

Several studies also investigated language impairments in bilingual individuals, and attempted to identify which treatments may be most beneficial for such patients. Bilingual patients with aphasia have been found to respond positively to conventional naming interventions,^6^ and to benefit more from monolingual than from bilingual interventions,^39,40^ especially when performed in the patient's non-native language.^40,43^ Interestingly, Kurland and Falcon^39^ revealed that training involving cognates, or words with similar semantic and phonological features in both of the languages spoken by the patient, may not necessarily contribute to the generalization of therapeutic benefits from one language to the other. These results suggest a possible interference effect, whereby the increased lexical access to words in one language may impair access to similar words in other languages. However, studies of bilingual patients with aphasia are still quite recent, and further investigations involving larger samples, more comprehensive assessments and longer follow-up periods must be performed to confirm these hypotheses.

One of the studies also compared the effects of conventional face-to-face therapy and computerized telerehabilitation.^32^ Such studies suggest a search for intervention models which are more accessible to patients who live far from rehabilitation centers or who have locomotor disabilities. The use of such strategies may also contribute to treatment adherence and to the generalization of treatment effects.

The effectiveness of *errorless and errorful learning*^49^ for the treatment of language impairments in patients with dementia were also compared by some of the studies reviewed. Although one study^37^ found errorless learning to be a superior treatment method, other investigations found no differences between the effectiveness of the two treatments.^5^ To settle these discrepancies, Class III studies must be performed to investigate the efficacy of each of these approaches.^50^

Although most pre- and post-treatment assessments involved language evaluation tools only, some studies investigated the effects of language interventions on cognitive skills, especially memory, attention, and the executive functions. These studies found that lexical-semantic interventions may lead to improvements in working memory and executive functions,^36^ and that functions such as inhibitory control may contribute to the generalization of therapeutic gains.^42^ These findings point to the presence of cross-domain effects of language stimulation on other cognitive abilities.

The pre- and post-treatment evaluation of cognitive abilities contributes to our comprehension of linguistic and cognitive functioning, and helps to elucidate which cognitive components may be involved in each method of lexical-semantic rehabilitation. This knowledge may help guide the planning of therapeutic interventions, and contribute to the prediction of patient prognosis and functional status following lexical-semantic therapy.

Although most of the studies analyzed produced positive results, their findings must be interpreted in light of a few limitations. The small sample size involved in the investigations limited the generalizability of their results. The variability in the tasks used to assess participant performance also prevented comparisons across studies and interventions. It is also important to note that most of the studies reviewed did not involve random assignment to treatment nor blinded pre and posttreatment assessments.

The generalization of therapeutic gains to untreated stimuli was seldom assessed in these studies, even though this variable is known to be an important indicator of the success of language interventions. In addition to evaluating performance on treated and untreated stimuli, intervention studies should also assess the effects of therapy on daily linguistic functioning.^48,51^ Questionnaires to evaluate improvements in the patient's daily routine following treatment may make especially important contributions in this regard, and should be more widely implemented given that restoring patient functioning can be considered the primary aim of rehabilitation programs. There is also a need for more extensive follow-up assessments after the end of treatment, so that the long-term benefits of each intervention can be identified and compared.^50^

The present review concluded that current research into lexical and semantic rehabilitation is still somewhat limited in its description of the procedures involved and the results obtained by different intervention strategies. There is a need for further studies which provide more detailed descriptions of lexical-semantic rehabilitation methods and their theoretical basis, so as to facilitate their replication by different investigators. Pre- and post-intervention assessments should also be similar across studies to allow for comparisons between investigations, and follow-up evaluations should be more carefully considered. Lastly, we suggest that the different lexical-semantic rehabilitation methods be evaluated through multicenter studies, which would allow for the participation of a larger sample.

One limitation of the present study was its exclusive focus on articles involving patients with dementia, TBI and stroke. Future reviews may also include articles discussing the effectiveness of lexical-semantic rehabilitation programs in patients with other neurological diseases such as multiple sclerosis, epilepsy, and cancer. Another limitation is the fact that the present review did not follow all PRISMA guidelines, since its scope did not include an evaluation of the quality of the studies. However, the present review did follow 20 out of the 27 items listed in the PRISMA checklist.^52^

The lexical-semantic interventions described tended to make use of decontextualized stimuli. Therefore, given the facilitating effects of context on word learning and lexical access, there is a need for greater investment in context-based lexical-semantic rehabilitation strategies. Such studies could contribute greatly to the evaluation of context effects on lexical-semantic skills.
